# A new species of *Stenasellus* Dollfus, 1897 from Iran, with a key to the western Asian species (Crustacea, Isopoda, Stenasellidae)

**DOI:** 10.3897/zookeys.766.23239

**Published:** 2018-06-13

**Authors:** Valiallah Khalaji-Pirbalouty, Yaser Fatemi, Mohammad Javad Malek-Hosseini, Matjaž Kuntner

**Affiliations:** 1 Department of Zoology, Faculty of Basic science, Shahrekord University, Shahrekord, Iran; 2 Young Researchers and Elite Club, Bandar Abbas Branch, Islamic Azad University, Bandar Abbas, Iran; 3 Evolutionary Zoology Laboratory, Department of Organisms and Ecosystems Research, National Institute of Biology, Ljubljana, Slovenia; 4 Evolutionary Zoology Laboratory, Biological Institute ZRC SAZU, Ljubljana, Slovenia; 5 Department of Biology, Biotechnical Faculty, University of Ljubljana, Ljubljana, Slovenia

**Keywords:** Iran, *Stenasellus*, Stenasellidae, Stygobitic, Tashan Cave

## Abstract

A new stenasellid isopod is described from Tashan Cave, Khuzestan Province, south-west Iran, belonging to the genus *Stenasellus* Dollfus, 1897. The first recorded species of Stenasellidae from Iran, *Stenasellus tashanicus* **sp. n.**, is diagnosed by the presence of antennae with a minute squama bearing paired, long, robust setae; a maxilliped endite with six coupling hooks; and slender appendix masculina with an acute apex. A revised generic diagnosis is provided with a key to the six known western Asian *Stenasellus* species.

## Introduction

The genus *Stenasellus* Dollfus, 1897, with approximately 37 nominal species (Boyko et al. 2008), is the largest genus in the family Stenasellidae Dudich, 1924. This genus was established with the description of *Stenasellus virei* Dollfus, 1897 from the subterranean water of Padirac, France. According to Malard et al. (2014) and Magniez and Rahmadi (2006), members of the genus occur from southern Europe (France, Italy, Spain, Portugal) to east Africa, (Kenya, Somalia), the Arabian Peninsula (Oman), and east Asia (Thailand, Cambodia, Sumatra, Java). To date, five species of the genus *Stenasellus* have been described from western Asia: *S. asiaticus* Birstein & Starostin, 1949 from a thermal brook in southern Turkmenistan, in addition to four species (*S. henryi* Magniez & Stock, 2000; *S. grafi* Magniez & Stock, 2000; *S. messanai* Magniez & Stock, 2000 and *S. vermeuleni* Magniez & Stock, 2000) were described from Oman. Stygobitic Isopoda in Iran are poorly known, with a single described species *Microcharon raffaellae* Pesce, 1979 of the family Lepidocharontidae Galassi & Bruce, 2016. This species was found in the subterranean water of Shahrekord, Chaharmahal Va Bakhteyari Province (Pesce 1979).

As species of *Stenasellus* were reported from southeastern Turkmenistan to the south-eastern corner of the Arabian Peninsula and east Africa (Somalia, Kenya and Oman), it was also expected to document their presence in the intervening geographical regions, such as Iran and Yemen. As reported here, our finding of the new stygobiont isopod species from the Iranian underground environment narrows the gap between these broad geographical areas.

## Materials and methods

**Figure 1.**
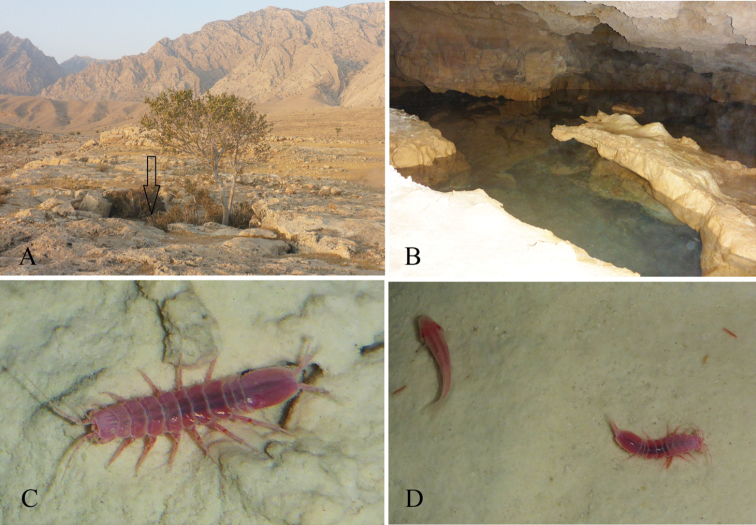
Tashan Cave. **A** Cave opening **B** a pool inside the cave **C** live specimen of *Stenasellus tashanicus* sp. n., in its habitat **D** *Stenasellus tashanicus* sp. n., and cave fish *Garra tashanensis* Mousavi-Sabet, Vatandoust, Fatemi & Eagderi, 2016.

Specimens for this study were collected from Tashan Cave, located inside a hill close to Sarjooshar Village, Tashan City, Behbahan County, Khuzestan Province, south-west Iran (Fig. 1A, B). The cave was visited seven times, but the isopods were only collected on the 13 and 27 August 2016. The specimens were preserved in 96% ethanol and deposited in the Zoological Museum, Shahrekord University, Iran. Appendages were drawn using an Olympus BX 51 compound microscope equipped with differential interference contrast and a camera lucida. Pencil drawings were scanned and electronically inked using Corel Draw X6 and were then processed using Adobe Photoshop CS5. Specimens were photographed with a Zeiss AxioCam ERc5s camera mounted on a Zeiss Stereomicroscope (Stemi 508). Appendages were dissected from specimens and stained by antibacterial glycerine-gelatine (Merck). The terminology of morphological characters follows Bruce and Buxton (2013).

Abbreviations: **ZMSU** – Zoological Museum, Shahrekord University, Iran; **RS** – robust seta/e; **SPS** – sensory palmate setae; **PMS** – plumose marginal setae.

## Taxonomy

### Aselloidea Latreille, 1802

#### Family Stenasellidae Dudich, 1924

##### Genus Stenasellus

Taxon classificationAnimaliaIsopodaStenasellidae

Dollfus, 1897

Stenasellus Dollfus, 1897:130; Racovitza 1924: 81; Birstein and Starostin 1949: 691; Magniez 1966: 177; Magniez 1968: 363; Magniez 1991: 99; Messana 1999: 1; Magniez and Stock 2000: 164.

###### Type species.

*Stenasellus virei* Dollfus, 1897, by monotypy.

###### Diagnosis.

Diagnoses to the genus can be found in Dollfus (1897) and Magniez (1966). The generic diagnosis presented here is more detailed than has been previously presented: Body lateral margins parallel and setose; pereonite VII longest; the antennal peduncle is 6-articulate, article VI longest, approximately 1.6 times the article V. Left mandible with incisor and lacinia mobilis bearing four cusps. Pereopod I with triangular carpus, dactylus elongated, an inferior margin with a row of contiguous scale-like flattened setae. Pereopods II-VII with an oval basis bearing some long distally plumose setae on the superior margin; dactylus shorter than elongated main unguis, bearing two secondary unguis. Pleopod I uniramous, protopod mesial margin with a simple RS or a single coupling hook, exopod elongated, mesial margin with a row distally plumose setae, distal margin fringed with a row of tiny simple short setae. Pleopod II exopod 2-articulate, article I short and without setae, article II longer than I, oval or round.

###### Remarks.

The first restrictive diagnosis to the genus was given by Dollfus (1897) when describing *Stenasellus virei* from the subterranean waters of France. Later, Magniez (1966) wrote a more detailed diagnosis, when comparing stenasellids of Africa. Subsequently, Magniez (1999) divided species of the genus *Stenasellus* of the Iberian Peninsula in two species groups: (*S. breuili* group and *S. virei* group). The species of *S. breuili* group being recognized by protopod of the male pleopod I with a single simple seta; pleopod II appendix masculina with cylindroid and elongated distal article, little or no twisted, bearing short setae (spine) on apical margin; and pleopods IV and V with endopod smaller than exopod with round apical margin. The species and subspecies that compose *S. virei* group stand out by the protopod of the male pleopod I with a single coupling hook on mesial margin, pleopod II appendix masculina fusiform distal article, more or less twisted, without apical short setae (spine); the exopods of pleopod IV and V, initially lamellar and broad subequal to apically rounded endopods.

In this work, two other groups of the genus *Stenasellus* are proposed: the first group that has pleopod III–V with entirely bilobed endopod are from western and south-eastern Asia: *S. bedosae* Magniez, 1991 and *S. brignolii* Pesce & Argano, 1981 (Thailand); *S. chapmani* Magniez, 1982 (Malaysia); *S. covillae* Magniez, 1987, *S. stocki* Magniez, 2001 and *S. strinatii* Magniez, 1991 (Sumatra); *S. grafi* Magniez & Stock, 2000; *S. henryi* Magniez & Stock, 2000 and *S. messanai* Magniez & Stock, 2000 (Oman). The species of the second group have pleopod II appendix masculina with slender, elongated, and tapering to an acute apex distal article and pleopod III–V with distally bilobed endopod. Except *S. cambodianus* Boutin & Magniez, 1985 from Cambodia, the remaining species of the second group are distributed in eastern Africa and western Asia: *S. kenyensis* Magniez, 1975 (Kenya), *S. costai* Lanza, Chelazzi & Messana, 1970 and *S. migiurtinicus* Messana, Chelazzi & Lanza, 1974 (Solalia); *S. vermeuleni* Magniez & Stock, 2000 (Oman), *S. tashanicus* sp. n. (Iran).

Based on descriptions and illustrations of the nominal species, there are some variations between the included species. The main variation is the shape of the pleopods I–V. The exopod of pleopod 1 is elongated and the medial margin of its protopod has a single coupling hook in most species (e.g., *S. virei* Dollfus, 1897; *S. strinatii* Magniez, 1991; *S. vermeuleni* Magniez & Stock, 2000), while some species have a pleopod 1 with short exopod and without any coupling hook (e.g., *S. grafi* Magniez & Stock, 2000; *S. stocki* Magniez, 2001). The second article of the pleopod II is round and possesses less than five marginal setae in some species (e.g., *S. henryi* Magniez & Stock, 2000; *S. grafi* Magniez & Stock, 2000; *S. nuragicus* Argano, 1968), whereas some species have an oval and elongated second article with more than 10 marginal setae (e.g., *S. vermeuleni* Magniez & Stock, 2000; *S. buili* Remy, 1949; *S. kenyensis* Magniez, 1975). Moreover, the endopod of pleopods III–V has a rounded distal margin (e.g., *S. virei* Dollfus, 1897; *S. asiaticus* Birstein & Starostin, 1949; *S. buili* Remy, 1949), some species have distally bifurcated endopod (e.g., *S. ruffoi* Messana, 1993; *S. vermeuleni* Magniez & Stock, 2000; *S. kenyensis* Magniez, 1975) and in some species the endopod is deeply bilobed (e.g., *S. javanicus* Magniez & Rahmadi, 2006; *S. grafi* Magniez & Stock, 2000; *S. henryi* Magniez & Stock, 2000). In addition, the medial margin of their maxilliped endite differs in having a different number of coupling hooks (2–6).

##### Stenasellus tashanicus sp. n.

Taxon classificationAnimaliaIsopodaStenasellidae

http://zoobank.org/F45E3E52-04AA-4238-8059-715858B80AD5

Figs 2 , 3 , 4

###### Material examined.

All material from Iran with locality data as follows.

***Holotype*.** ♂ (17.5 mm), Tashan Cave, Sarjooshar Village, Tashan City, Behbahan County, Khuzestan Province, Iran, 13 August 2016, 30°51'54"N, 50°10'29"E (altitude 559 m a.s.l.), coll. Fatemi, Y. (ZMSU 2010).

***Paratypes.*** 3 ♂♂ (17.1, 12.5, 12 mm), 2 ♀♀ (20, 11mm); 2 juveniles (7.3, 8.5 mm), same data as holotype (ZMSU 2011). 1 ♂ (15 mm), 1 ♀ (18 mm); 1 juveniles (5.5 mm), Tashan Cave, Sarjooshar Village, Tashan City, Behbahan County, Khuzestan Province, Iran, 27 August 2016, 30°51'54"N, 50°10'29"E (altitude 559 m a.s.l.), coll. Fatemi, Y. and Malek-Hosseini, M.J. (ZMSU 2012).

###### Diagnosis.

Body dorsal surface smooth, with scattered marginal setae. Antenna reaching to pereonite V posterior margin in male specimen, with a squama bearing three simple setae on the outer margin of the third article. Maxilla lateral and middle endites each bearing 11 curved pectinate RS; mesial margin of maxilliped endite with six coupling hooks. Appendix masculina slender, elongated, tapering to a curved acute apex; endopod of pleopods III–V distally bifurcated.

###### Description of male.

**Figure 2.**
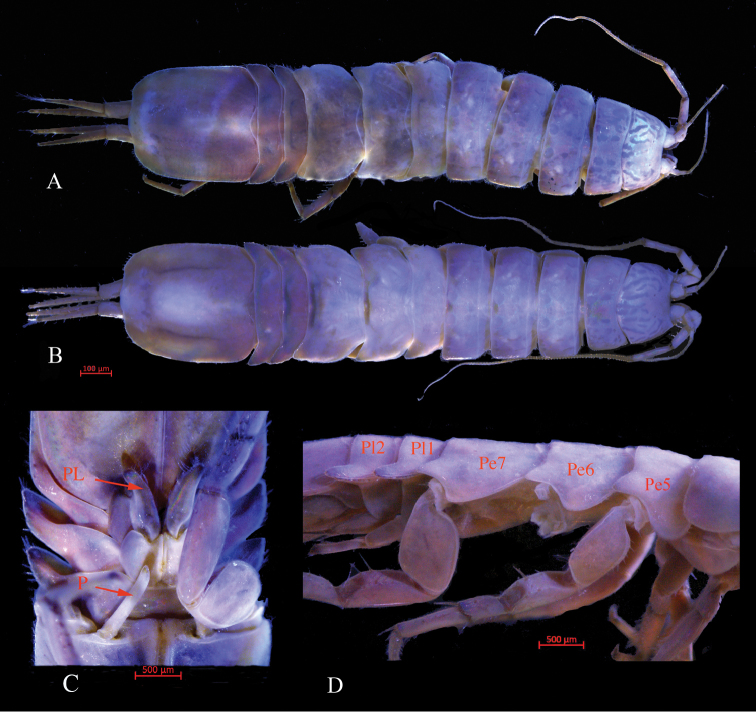
*Stenasellus tashanicus* sp. n., **A** holotype (ZMSU 2010) dorsal view **B** female dorsal view **C** holotype, ventral view (P: Penes, PL: Pleopod I) **D** lateral view (Pl1, Pl2: Pleonites I & II; Pe5, Pe6, Pe7: Pereonites V–VII).

*Body* completely coral pink in the live specimen (Fig. 1C, D), length 4.2 as greatest width, *head* trapezoidal, with slightly concave frontal margin, dorsal surface smooth. Pereonites II–IV subequal in length, with rounded lateral margins, pereonites V–VII with posterolateral margins projected posteriorly, pereonites VI and VII sub-equal, longest (Fig. 2A, D). Pleonites I–II subequal in length, with projected posterolateral corner.

*Pleotelson* elongated, 1.4 as long as broad, posterior margin with two slight excavations; with scattered marginal setae.

**Figure 3.**
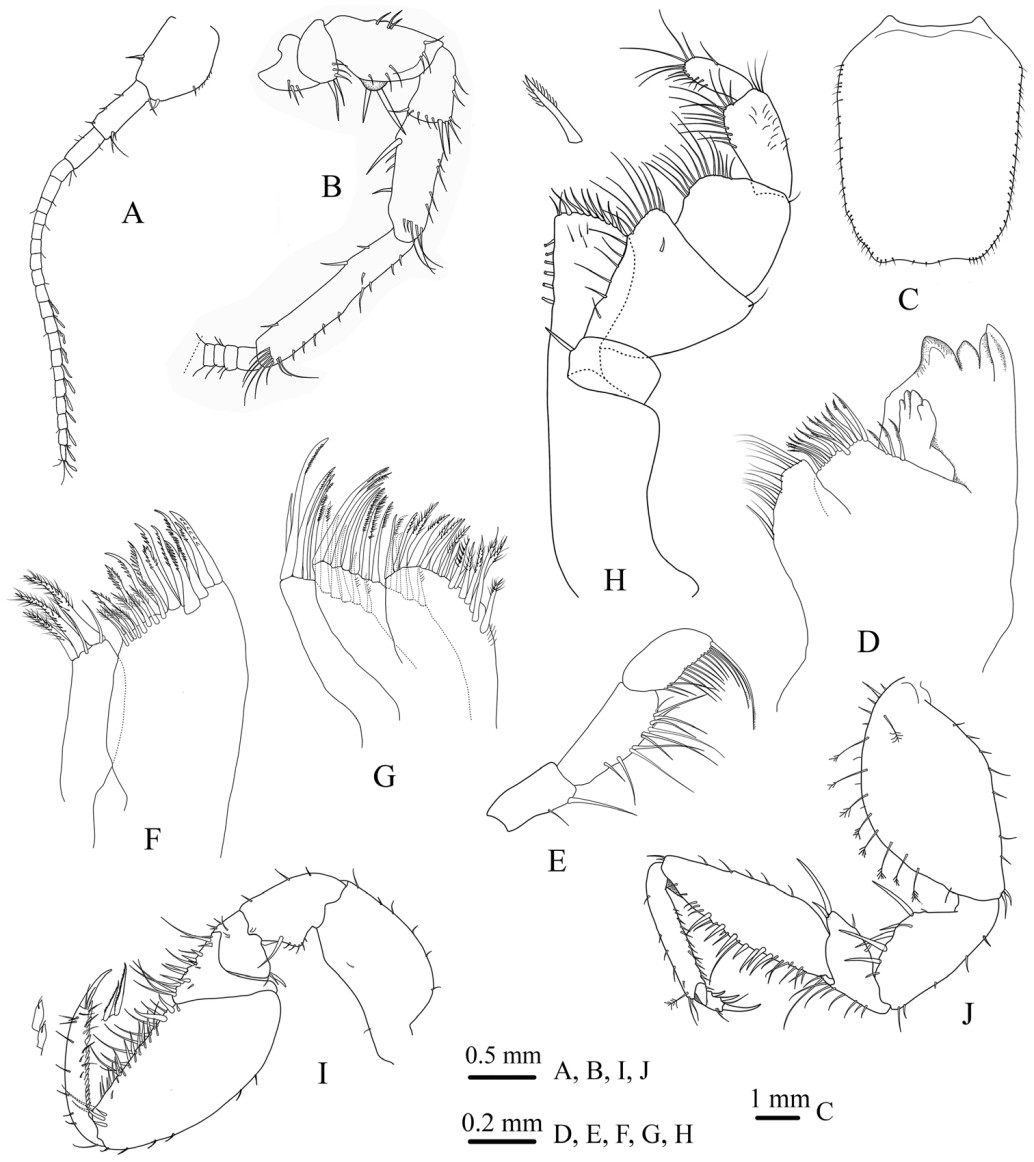
*Stenasellus tashanicus* sp. n., holotype (ZMSU 2010) **A** antennula **B** antenna **C** pleotelson **D** left mandible **E** palp of mandible **F** maxillula **G** maxilla **H** maxilliped **I** pereopod I **J** pereopod II.

*Antennula* (Fig. 3A) short, reaching pereonite I posterior margin, slightly longer than the peduncle of antenna, flagellum with 18 articles, articles 8–18 each bearing a single aesthetasc.

*Antenna* (Fig. 3B) peduncle articles I and II reduced; the four others longer, increasing in length from the fourth to the sixth; article VI about 1.6 times as article V, with long simple setae distally; article III with minute squama bearing two long RS, flagellum reaching to pereonite V posterior margin, up to 86 articles.

*Left mandible* (Fig. 3D, E) incisor and lacinia mobilis with four cusps, spine row of 18 serrate spines, molar with a row of long, tiny, simple setae. Palp article II longer than I, articles III distolateral margins with approximately 15 pectinated setae.

*Maxillula* (Fig. 3F) lateral endite apical margin with 12 serrate RS and eight tiny serrated smaller setae; mesial endite with three long, robust, comb and two short simple setae.

*Maxilla* (Fig. 3G) lateral and middle endites each with 11 curved pectinate RS; mesial endite with several rarely plumose, long robust combs, and slender simple setae.

*Maxilliped* (Fig. 3H) endite mesial margin with six coupling hooks, distal margin with approximately 10 serrated and rarely plumose RS; palp article I with single RS on the inferior margin, palp articles II–V with several long simple setae on the inferior margin.

*Pereopod I* (Fig. 3I) basis length 1.66 width, ischium superior margin with one RS on distal corner and five small RS on the medial projection; merus supradistal angle with three RS; carpus triangular, inferior margin covered with several long and short simple RS; propodus inferior margin covered with several long simple RS set in amongst some serrated RS; dactylus 9.2 times as long as basal width, inferior margin with a row of contiguous scale-like flattened setae with accessory setulae, main unguis elongate.

*Pereopod II* (Fig. 3J) basis about 1.8 times as long as the greatest width, superior margin with nine long distally plumose setae; ischium superior margin with five long RS; merus supradistal angle with two long RS, inferior margin with nine long simple setae; carpus superior margin with five simple setae, inferior margin covered with several long and short simple RS; propodus inferior margin covered with several short, simple, acute setae, supradistal angle with two long simple and single sensory palmate setae; dactylus shorter than main unguis, with two secondary unguis.

**Figure 4.**
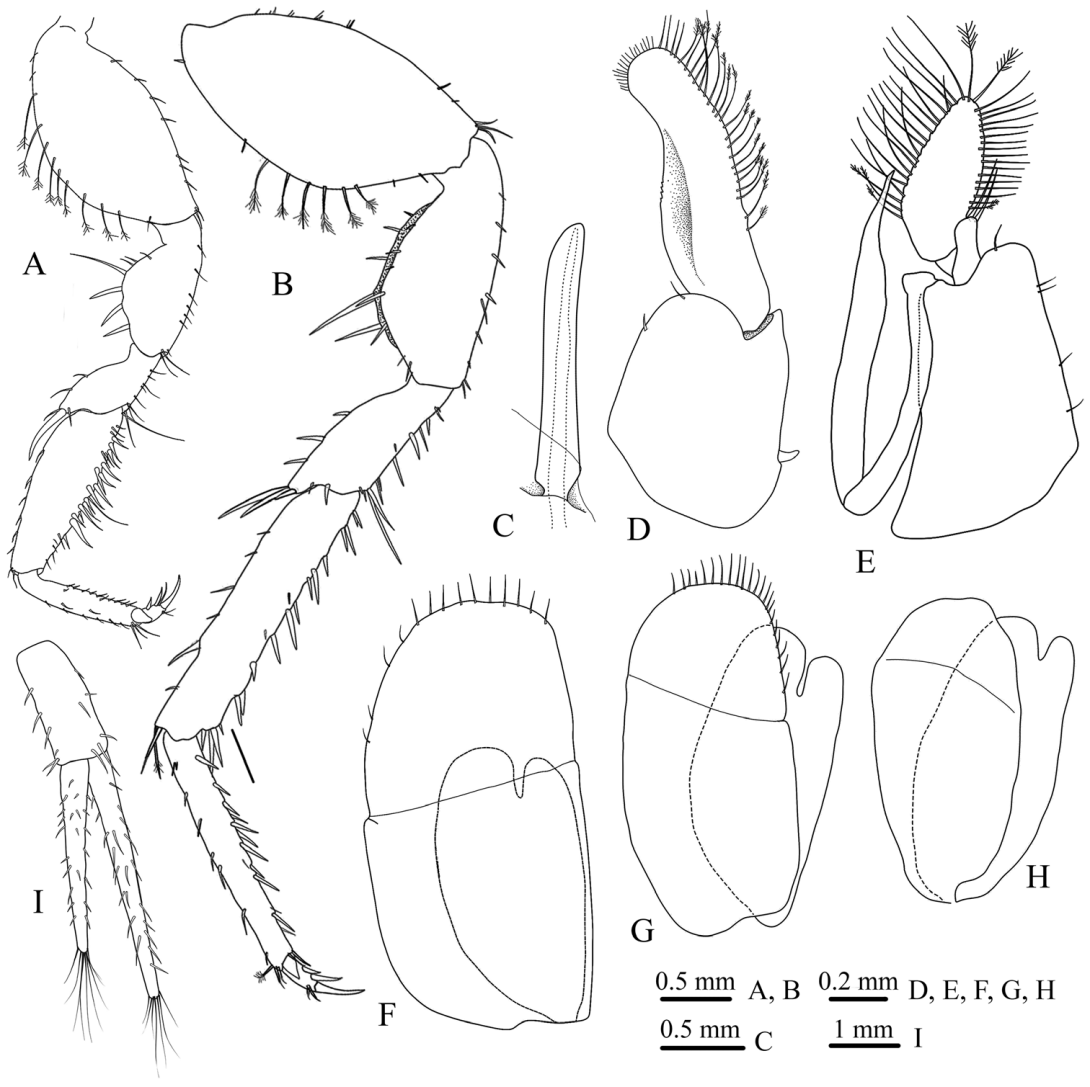
*Stenasellus tashanicus* sp. n., holotype (ZMSU 2010) **A** pereopod III **B** pereopod VII **C** Penial processes (left ramous) **D–H** pleopods I–V **I** Uropod.

*Pereopod III* (Fig. 4A) is similar to pereopod II as illustrated.

*Pereopod VII* (Fig. 4B) basis about two times as long as the greatest width, superior margin with nine long distally plumose setae; *Ischium* length 2.2 width; merus supradistal angle with three long RS; carpus length 5.0 width, inferior margin covered with several long and short simple RS, supradistal angle with a long simple and a single sensory palmate setae; propodus length 7.3 width, inferior and superior margins covered with several short, simple, acute setae, supradistal angle with two long simple and a single sensory palmate setae; dactylus with elongated main unguis, bearing two secondary unguis.

*Penial processes* (Figs 2C, 4C) elongated, cylindrical, about 5.8 times as long as the greatest width.

*Pleopod I* (Fig. 4D) protopod length 1.2 width, mesial margin with a single coupling hook, exopod elongated, mesial margin with a row of 21 PMS and four simple setae, apical margin with row of ~18 simple fine setae, lateral margin concave.

*Pleopod II* (Fig. 4E) protopod elongated, length 1.7 width, exopod article I small, without seta, article II oval, with ~ 41 PMS; endopod small, with two apical, long, simple setae; *appendix masculina* length 1.4 article I length, 11.4 basal length, tapering to curve acute apex.

*Pleopod III* (Fig. 4F) exopod with transverse suture, apical margin with ten slender simple setae; endopod 0.6 as long as exopod, bifurcated distally.

*Pleopod IV* (Fig. 4G) exopod with transverse suture, distolateral margin with 22 slender simple setae; endopod 0.8 as long as exopod, bifurcated distally.

*Pleopod V* (Fig. 4H) exopod and endopod subequal in length, without marginal setae.

*Uropods* (Fig. 4I) protopod and rami covered with scattered acute simple setae; endopod longer than exopod, both rami with distal tuft of setae.

###### Female.

(Fig. 2B) Typically, longer than the male, apart from sexual characteristics similar to male, pleotelson is broader and antenna is longer than male.

###### Etymology.

The name of this species comes from the type locality, the Tashan Cave, Iran.

###### Habitat.

The isopods were collected from two pools in the dark zone of the Tashan Cave (at 20 to 200 cm depths). They were observed in all life cycle stages. They were observed crawling on the floor and hiding inside the sediment and cavities of the pools, as well as swimming in the water column. Mousavi-Sabet et al. (2016) described a blind fish from this cave (see Fig. 1D).

###### Remarks.

*Stenasellus tashanicus* sp. n. can be identified by a slender and distally acute appendix masculina, and a maxilliped endite with six coupling hooks on the mesial margin. The new species is the largest known *Stenasellus* member: length up to 18 mm in males and 20 mm in females. The new species is similar to *S. vermeuleni* Magniez & Stock, 2000 (known from Wadi Halban, Oman), in having an appendix masculinum with acute apex. The shape of pleopods III–V in both species is also similar. Based on the drawings and description of *S. vermeuleni*, the new species differs by having a uropodal exopod smaller than the endopod (rather than subequal in length), pleopod I apical margin with a row of 18 simple fine setae (rather than six), exopodal article II of pleopod II is narrower than pleopod II in *S. vermeuleni* and pleopod V exopod and endopod subequal in length (rather than a smaller exopod). Based on the description and drawings of *S. asiaticus* by Birstein and Starostin (1949) from Turkmenistan, this species is readily distinguished from the new species by setose body dorsal surface (rather than smooth body surface) and its flattened appendix masculina (rather than a narrow with an acute apex).

### Key to the Western Asian species of *Stenasellus*

**Table:** 

1	Body dorsal surface setose; pleopod endopod of pleopods III–V distally monolobate	***S. asiaticus*** (Turkmenistan)
–	Body dorsal surface smooth; pleopod endopod of pleopods III–V distally bilobate	**2**
2	Pleopod II exopod article II small, with less than five marginal setae; Appendix masculina flat and swollen	**3**
–	Pleopod II exopod article II large, with more than eight marginal setae; Appendix masculina elongate distally acute	**4**
3	Pleopod II protopod heart shaped	***S. grafi*** (Oman)
–	Pleopod II protopod trapezoid shaped	***S. henryi*** (Oman)
4	Appendix masculine dislaolateral margin fringed with tiny setae	***S. messanai*** (Oman)
–	Appendix masculine dislaolateral margin without setae	**5**
5	Antenna squama with three robust setae, exopod of pleopod I apical margin with a raw of ~6 simple fine setae; pleopod II exopod about 1.4 times as long as greatest width	***S. vermeuleni*** (Oman)
–	Antenna squama with II robust setae, exopod of pleopod I apical margin with a raw of ~18 simple fine setae; pleopod II exopod about 2.4 times as long as greatest width	***S. tashanicus* sp. n.**

## Supplementary Material

XML Treatment for Stenasellus

XML Treatment for Stenasellus tashanicus
